# Variation of Procalcitonin Level in Elective Cardiac Surgery at a Tertiary Cardiac Centre: A Prospective Longitudinal Study

**DOI:** 10.1002/hsr2.72574

**Published:** 2026-05-28

**Authors:** Prati Badan Dangol, Battu Kumar Shrestha, Suraksha Dhungana, Gaurab Mainali, Madanika Ghimire, Amrita Pandey

**Affiliations:** ^1^ Department of Nursing Shahid Gangalal National Heart Centre Kathmandu Nepal; ^2^ Department of Anesthesiology Shahid Gangalal National Heart Centre Kathmandu Nepal; ^3^ Shahid Gangalal National Heart Centre Kathmandu Nepal; ^4^ Nepalese Army Institute of Health Sciences Kathmandu Nepal

**Keywords:** delayed complication, elective cardiac surgery, infection, mortality, procalcitonin

## Abstract

**Background:**

Procalcitonin (PCT) levels often rise after cardiac surgery due to an ongoing postoperative inflammatory response. The changes in PCT levels are useful for the early detection of postoperative infections. We assessed the variation of procalcitonin levels in patients undergoing elective cardiac surgery at the leading tertiary cardiac centre to support clinical decision‐making and future studies in our setting.

**Methods:**

An observational longitudinal study was conducted among 273 adults who underwent elective cardiac surgery. Patients with perioperative signs of infections, systemic infections, and use of corticosteroids within the last 7 days were excluded. SPSS version 26 was used for data analysis. We measured baseline serum PCT levels before surgery, and on days 1, 3, and 5 after surgery. A *p*‐value < 0.05 was considered statistically significant.

**Results:**

Our study showed a characteristic double peak in procalcitonin levels. The baseline PCT level was 0.10 ng/mL. Postoperatively, the median PCT values were 11.9 ng/mL on day 1, 6.8 ng/mL on day 3, and 15.9 ng/mL on day 5. The Friedman test showed a statistically significant difference in the levels of PCT at the four time points (*χ* ^2^ (3) = 417.721, *p* < 0.001). Kendall's coefficient of concordance (W = 0.529) indicated moderate to strong agreement in this trend. Post hoc Wilcoxon Signed‐Rank test with Bonferroni correction confirmed a significant rise on day 1, a decline by day 3, and a subsequent increase on day 5.

**Conclusion:**

There was a dynamic postoperative variation in levels of PCT. A secondary rise in PCT levels may indicate the development of delayed complications like Surgical Site Infections and Sepsis requiring clinical attention. Therefore, monitoring serial PCT trends over time is more important than reliance on isolated values.

## 1 Introduction and Background

Cardiac surgery triggers an acute inflammatory response as a result of surgical trauma and exposure of blood to a non‐physiological surface. This may subsequently develop into systemic inflammatory response syndrome (SIRS), which mimics the signs of postoperative infections [1, 2]. Identification of infectious complications in these patients is typically challenging [3].

Procalcitonin (PCT) has become increasingly popular as a useful biomarker of inflammation. PCT is a peptide precursor of calcitonin released from C‐cells of the thyroid gland [2, 3]. Normal plasma concentration of PCT is below 0.1 ng/mL. Procalcitonin (PCT) helps assess bacterial infection severity based on its level in the blood. Values < 0.05 ng/mL are normal, while 0.05–0.5 ng/mL suggests low risk of systemic infection. Levels of 0.5–2.0 ng/mL indicate possible bacterial infection, 2.0–10.0 ng/mL suggest likely sepsis, and > 10.0 ng/mL point to severe sepsis or septic shock. However, PCT may also rise after surgery or trauma without infection, so clinical context is essential. Adapted from Meisner et al. [4]. The rise in PCT levels after cardiac surgeries has been associated with complement activation, cytokine release, elevated vasopressin levels, and endotoxemia as a result of SIRS [5]. In uncomplicated cases, the values of PCT peak within 24 h postoperatively and return to baseline within 5 days of surgery. However, in the presence of infection, the levels continue to stay elevated beyond 1 week [3]. Under such circumstances, other organs, such as the lungs, liver, and intestine, act as new sources of procalcitonin production [6].

PCT levels are also increased in non‐infectious causes, reportedly SIRS without infections, such as trauma, burns, and heat stroke [7]. However, the rise in serum PCT is greater in bacterial infections than in other noninfectious causes [6]. PCT levels are found to be more sensitive and more specific than CRP levels for differentiating bacterial from noninfective causes of inflammation [8]. PCT shows an earlier rise than C‐reactive protein (CRP), with a rapid peak after infection. If the response to the treatment is good, its level also returns to the normal range faster than CRP. This shows PCT is potentially a better biomarker for sepsis [9].

The changes in procalcitonin levels after elective cardiac surgery are useful for early diagnosis of postoperative infections. However, there are limited data regarding the postoperative dynamics of procalcitonin in the context of Nepal. Most of the available data are from the research conducted in an international scenario. They may not always be applicable in our setting, considering the differences in infrastructure and clinical practices. Therefore, we aim to address this crucial research gap by analysing the variation of procalcitonin levels after elective cardiac surgery in the leading tertiary cardiac centre of Nepal. Our findings are expected to be useful in clinical decision‐making for the early management of postoperative infections. The context‐specific reference data from this study can be relevant for future studies in our setting.

## 2 Methodology

### 2.1 Ethical Consideration

Ethical clearance was obtained from the Institutional Review Committee (IRC). Informed consent was taken from the patients or the relatives.

### 2.2 Study Design and Sample Collection

A prospective longitudinal study was conducted at Shahid Gangalal National Heart Centre (SGNHC), a tertiary cardiac Centre in Nepal, after obtaining ethical approval from the Institutional Review Committee, SGNHC (IRC No: 3‐2023). An informed written consent was taken from respondents then data was taken in 3–5 settings from the respondents. A first interview was taken prior to surgery in the preoperative ward; then data was also taken on post‐operative Day 1, 3, 5, and on the day of discharge. Data was collected from February 2023 to July 2024. A non‐probability, purposive sampling technique was used. A total of 273 participants who had undergone elective cardiac surgery were included. Validity of the questionnaire was ensured by infection prevention and control experts of the centre.

### 2.3 Study Population

The study included adult patients ( ≥ 18 years) undergoing elective cardiac surgery. Patients were excluded if they had preoperative or perioperative evidence of infection, systemic inflammatory or infectious conditions, recent corticosteroid use (within 7 days), emergency surgery, or incomplete data on PCT measurements.

### 2.4 Data Analysis

Quantitative data were tested for normality using the Shapiro–Wilk test. Variables that were normally distributed are presented as mean and standard deviation, while those that did not follow a normal distribution are reported as median and interquartile range. Qualitative data are expressed as proportions and percentages. Since the data were paired, paired tests were performed. Normality and sphericity assumptions were checked. As most data violated these assumptions, the non‐parametric Friedman test was used for analysis. When the Friedman test indicated statistical significance, post hoc analysis was conducted using the Wilcoxon signed‐rank test with Bonferroni correction. A *p*‐value less than 0.05 was considered statistically significant. Data were analysed using SPSS version 26.

## 3 Results

Table 1 shows that this study included 273 adult patients who underwent cardiac surgery. The average age was 46.5 years, with a fairly wide spread ( ± 14.26 years), and the gender distribution was almost equal; 48.4% were male and 51.6% were female.

**Table 1:** Baseline characteristics of the study population.

Baseline variables	Value
Age	46.53 ± 14.26 years
Gender	
Male	132 (48.4%)
Female	141 (51.6%)
Systolic Blood Pressure	110 [100–124] mm of Hg
Diastolic Blood Pressure	70 [60–75] mm of Hg
Respiratory Rate	20 [18–22] breaths/minute
Temperature	97 [97–97.6] °F
Heart Rate	78 [72–82] beats/minute
CRP	0.50 [0.50–5] mg/L
PCT	0.1 [0.05–0.1] ng/mL
TC	7210 [5745–8000] cells/mm^3^
Comorbidity	
None	223 (81.7%)
Systemic Hypertension	16 (5.9%
Diabetes Mellitus	20 (7.3%)
Bronchial asthma	2 (0.7%)
Diabetes Mellitus and Hypertension	1 (0.4%)
Others	11 (4%)
Types of Surgery	
Valvular Surgery	226 (82.8%)
CABG	31 (11.4%)
Valve and CABG	10 (3.7%)
Others	6 (2.2%)

At the time of admission, most patients had stable vital signs. The median systolic blood pressure was 110 mmHg (interquartile range [IQR]: 100–124), and the median diastolic pressure was 70 mmHg (IQR: 60–75). The median respiratory rate was 20 breaths per minute, and the heart rate was 78 beats per minute, both within expected ranges for non‐critically ill patients. Body temperature showed minimal variation, with a median of 97.0 °F (IQR: 97.0–97.6).

Baseline inflammatory markers were also mostly within normal limits. The median CRP level was 0.50 mg/L, and most patients fell within a narrow range (IQR: 0.50–5.00). Procalcitonin (PCT), another marker of systemic inflammation and infection, had a very low baseline median of 0.10 ng/mL (IQR: 0.05–0.10), suggesting that nearly all patients began their hospital course without evidence of significant infection. The total leucocyte count had a median of 7210 cells/mm^3^ (IQR: 5745–8000), staying within the normal reference range.

Most patients (81.7%) had no chronic comorbid conditions. Among the remainder, diabetes mellitus (7.3%) and hypertension (5.9%) were the most common. Only a small number had both diabetes and hypertension (0.4%), asthma (0.7%), or other conditions (4%).

Regarding the types of cardiac surgeries performed, valvular surgery was the most prevalent, accounting for 82.8% of all cases. Another 11.4% of patients underwent coronary artery bypass grafting (CABG), and 3.7% had a combination of valve surgery and CABG. A small group (2.2%) had other surgical procedures not categorized separately due to low numbers.

**Figure 1:**
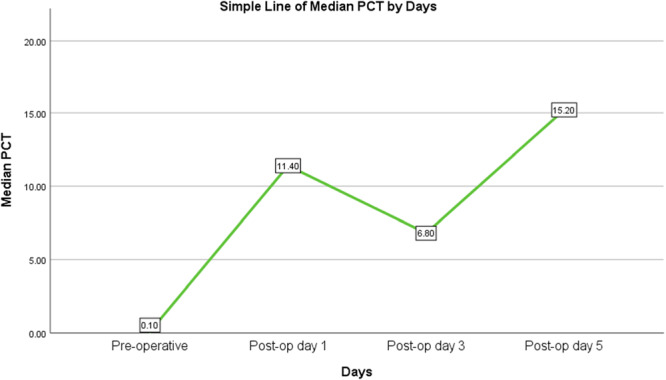
A line chart showing the trend of change of PCT across time.

Table 2 and Figure 1 shows pre‐operatively, the median procalcitonin level was low at 0.1 ng/mL (IQR: 0.05–0.1), indicating no evidence of infections. On postoperative Day 1, there was a marked rise to 11.4 ng/mL (IQR: 2.7–29.1), reflecting an acute inflammatory response to surgical stress. By Day 3, levels decreased to 6.8 ng/mL (IQR: 1.7–17.35), suggesting partial recovery or resolution of the initial response. However, by Day 5, the median PCT increased again to 15.2 ng/mL (IQR: 5.4–32.05), which may indicate ongoing inflammation, sepsis, or surgical site infection requiring clinical attention.

**Table 2:** Trend of changes of level of PCT over time.

Time	Value
Pre‐operative PCT	0.1 [0.05–0.1] ng/mL
Day 1 PCT	11.4 [2.7–29.10] ng/mL
Day 3 PCT	6.8 [1.7–17.35] ng/mL
Day 5 PCT	15.2 [5.4–32.05] ng/mL

In Table 3, the Friedman test revealed a statistically significant difference in PCT levels across the four time points (*χ* ^2^ (3) = 417.721, *p* < 0.001). The Kendall's coefficient of concordance (W = 0.529) indicates a moderate to strong agreement in the ranks across time.

**Table 3:** Statistical tests that were applied to assess changes in procalcitonin levels from the preoperative baseline across multiple postoperative days.

Test	Chi‐square	Df	*p* value	Kendall's W
Friedman test	417.721	3	< 0.001	0.529

In Table 4 Paired comparisons of procalcitonin (PCT) levels across perioperative time points were conducted using the Wilcoxon signed‐rank test, with Bonferroni correction applied for multiple comparisons (adjusted significance threshold = 0.0125).

**Table 4:** Post hoc pairwise comparisons (Wilcoxon Signed‐Rank test with Bonferroni correction).

Comparison	Median Difference	Wilcoxon Z	Adjusted *p*‐value
Day1 vs Preop	10.80 (2.15 to 28.30)	−13.39	< 0.001
Day 3 vs Day 1	−2.20 (−13.30 to 0.80)	−6.14	< 0.001
Day 5 vs Day 1	2.60 (−13.30 to 17.20)	−1.25	0.840
Day 5 vs Day 3	6.40 (−4.92 to 19.8)	−5.353	< 0.001

A significant increase in PCT was observed from preoperative levels to postoperative Day 1, with a median difference of 10.80 ng/mL (95% CI: 2.15 to 28.30; Z = −13.39; adjusted *p* < 0.001). Between Day 1 and Day 3, PCT levels significantly declined (median difference: −2.20 ng/mL; 95% CI: −13.30 to 0.80; Z = –6.14; adjusted *p* < 0.001). No statistically significant change was noted between Day 1 and Day 5 (median difference: 2.60 ng/mL; 95% CI: −13.30 to 17.20; Z = −1.25; adjusted *p* = 0.840). However, a significant increase in PCT was observed from Day 3 to Day 5 (median difference: 6.40 ng/mL; 95% CI: −4.92 to 19.80; Z = −5.35; adjusted *p* < 0.001).

## 4 Discussion

This study showed a distinct triphasic trend with a double peak of procalcitonin level. An elevation was observed on postoperative day 1, with a median value of 11.9 ng/mL, followed by a decline to 6.8 ng/mL on day 3. Notably, PCT levels rose again on day 5, reaching a median of 15.9 ng/mL. Statistical analysis using the Friedman test demonstrated a significant variation in PCT levels across the pre‐and postoperative time points (*p* < 0.001). The Kendall coefficient of concordance (W = 0.529) indicated a moderate to strong agreement in this trend. Pairwise comparisons using the Wilcoxon signed‐rank test with Bonferroni correction confirmed a significant increase from baseline to day 1, a significant decline from day 1 to day 3, and a subsequent significant increase on day 5 compared to day 3.

Our finding of the initial rise of procalcitonin aligns with established evidence highlighting the elevation of PCT level in immediate postoperative days [1, 3, 10, 11, 12]. A similar study conducted by Meisner et al. reports an increase in PCT levels above the normal limit in 59% of patients undergoing cardiac and thoracic surgery [10]. The initial rise in PCT is attributed to surgical procedure, which leads to varying degrees of tissue inflammation and activation of cytokines secondary to tissue trauma, leading to systemic response by the body's inflammatory system, known as Systemic Inflammatory Response Syndrome (SIRS) [3, 5, 10, 12, 13].

A systematic review of literature conducted by Christoph Sponholz et al. showed that in uncomplicated cardiac surgery, PCT level rises and peaks within 24 h postoperatively and returns to baseline within the first week [3]. This result is also supported by other studies conducted in different settings [10, 11, 12, 14].

Procalcitonin is a useful marker of inflammation whose value increases in both infectious and noninfectious conditions [15, 16]. However, patients with infection show higher PCT levels. The values are unlikely to exceed 5 ng/ml in uncomplicated cases [3, 17]. Another research conducted by Matthias Klingele et al. showed that patients with elevated PCT levels are at higher risk of developing delayed complications despite an initially uneventful postoperative course [11]. This finding is also supported by other studies, which showed an increase in PCT level in 1st POD in patients developing delayed complications like SIRS, Sepsis, myocardial infarction, bloodstream infection, respiratory failure, renal failure, and cardiac failure compared to patients without delayed complications [3, 12, 14, 15].

A correlation between the type of surgery and the degree of PCT elevation has been reported by multiple studies [3, 5, 13, 18]. A study by Loebe et al. showed that the rise in PCT is more after valvular and aortic surgery compared to CABG. PCT levels were greater than or equal to 5 ng/mL in 39% and 35% of patients undergoing valvular and aortic surgery, respectively, compared to 13% of patients who underwent CABG [5]. Intraoperative parameters also affect the evolution of PCT postoperatively, e.g., duration of surgery [17], duration of CPB [13, 16], and aortic cross‐clamping time [16]. The rise in PCT is more after major operations like thoracic, abdominal, and vascular surgeries compared to patients who have undergone minor, aseptic surgeries [17].

In our study, there is a secondary rise in serum PCT after the initial decline, which is noteworthy. Comparable patterns have been observed and explained by other studies as well. A systematic review of literature by Cristoph Sponholz et al. highlights the importance of the study of the dynamics of PCT rather than relying on absolute values. In the absence of complications, the PCT level peaks in the 1st POD, followed by a gradual decline, whereas it remains elevated in the presence of complications like infection [3]. Another similar study by Serdar Celebi et al. in a paediatric population undergoing open heart surgery revealed a postoperative double peak procalcitonin curve in patients with SIRS plus organ failure during ICU stay [16]. Likewise, a study in the paediatric population by Li Xia et al. showed a similar pattern of procalcitonin kinetics in both groups of patients developing and not developing infection till the 3rd POD. A secondary rise of procalcitonin from days 4 and 7 was positively correlated with the development of infection [19]. A study by Mathieu Desmard et al. in lung transplantation patients also demonstrated that doubling of plasma PCT after initial decline was significantly associated with postoperative respiratory tract infection [20].

Though the levels of procalcitonin for the prediction of infection are known, different values are suggested by different studies. A meta‐analysis by Christina Wacker et al. suggested PCT as an important marker of postoperative complications with a sensitivity of 77% and specificity of 79% [21]. Similarly, a prospective cohort study conducted by Matthias Klingele et al. showed that patients with PCT levels above the cutoff value of 2.95 ng/mL on the first postoperative day had a 66 times higher risk of developing delayed complications compared to those with PCT levels below this threshold [11]. Correlating with our study, where on the first postoperative day, the median value of PCT was 11.9 ng/mL, which later rose to a median of 15.9 ng/mL on the 5th POD, indicating the probability of delayed complications, most commonly sepsis.

Conversely, the utility of PCT as a standalone diagnostic marker is debated. A study conducted by Chakravarty Murali et al. in 819 patients who underwent cardiothoracic surgery failed to elicit a positive correlation between elevated PCT levels and postoperative infection, with a sensitivity of 50% and a specificity of 17%. The positive predictive value was 12%, suggesting elevated PCT level alone should not be used for diagnosing sepsis [22]. This finding is supported by another study by F. kalay et al. in 91 elective cardiac surgery patients, suggesting its value can be altered by many other factors, including infection [1]. These findings highlight the limitations of relying solely on absolute PCT values for diagnosing postoperative infection. More sensitive detection methods, such as the enzyme‐linked fluorescent assay (ELFA), have been developed to improve the accuracy of PCT measurement. ELFA has a functional sensitivity of approximately 0.09 ng/mL, which is close to the upper normal limit, allowing earlier and more precise detection of subtle increases in PCT levels [23].

Large randomized controlled trials have further evaluated the clinical utility of procalcitonin‐guided strategies in antimicrobial stewardship. The Stop Antibiotics on Procalcitonin guidance Study (SAPS) demonstrated that PCT‐guided algorithms can safely reduce antibiotic exposure, particularly in healthcare settings with relatively lower baseline antibiotic use, without adversely affecting patient outcomes [24]. The SISPCT trial (Sodium Selenite and Procalcitonin Guided Antimicrobial Therapy in Severe Sepsis) was a large‐scale, multicentre randomized clinical trial conducted in Germany to evaluate two different interventions for patients with severe sepsis or septic shock. One arm of the study specifically assessed whether a procalcitonin‐guided strategy for antimicrobial therapy could improve clinical outcomes and optimize antibiotic use in critically ill septic patients. Although the trial did not demonstrate a statistically significant reduction in 28‐day mortality with PCT‐guided therapy, it showed that serial PCT monitoring could support earlier discontinuation of antibiotics without increasing adverse outcomes, thereby contributing to antimicrobial stewardship practices [25].

The PRORATA trial (PROcalcitonin to Reduce Antibiotic Treatments in Acutely ill patients) was a landmark multicentre, randomized controlled trial published in The Lancet in 2010. It aimed to determine whether a procalcitonin‐guided algorithm could safely reduce antibiotic exposure among critically ill patients admitted to the Intensive Care Unit (ICU) with suspected bacterial infections. The study demonstrated that PCT‐guided management significantly reduced the duration and overall exposure to antibiotics while maintaining similar mortality and treatment success rates compared with standard care. These findings established PCT as a useful adjunct for antimicrobial stewardship, particularly in reducing unnecessary antibiotic use in critically ill populations [26].

Advances in assay technology have further enhanced the clinical applicability of procalcitonin measurement. The development of highly sensitive automated assays, such as the BRAHMS PCT Kryptor®, which was among the first to receive regulatory approval for use in severe sepsis and septic shock, has improved the accuracy and turnaround time of PCT estimation (23). Subsequent adaptations across various diagnostic platforms, utilizing immunoluminometric, enzyme‐linked immunofluorescent, and chemiluminescent techniques, have allowed broader implementation in clinical practice, although minor variations in analytical performance persist.

## 5 Limitations

A key limitation of this study is the absence of cardiac surgical risk stratification parameters (e.g., EuroSCORE II) and detailed intraoperative variables such as cardiopulmonary bypass and aortic cross‐clamp times. As the study was designed to describe postoperative PCT patterns rather than analyse determinants or outcomes, these variables were not systematically collected. Consequently, the influence of surgical complexity and intraoperative factors on PCT variation could not be evaluated.

## 6 Conclusion

Our findings showed postoperative triphasic variation of PCT level. A secondary rise in PCT levels may indicate the development of delayed complications like sepsis requiring clinical attention. This study highlights the importance of serial PCT monitoring over reliance on isolated values. An early identification of delayed postoperative complications in patients undergoing elective cardiac surgery can be an important marker to guide clinical assessment, initiation of treatment, and improvement in overall patient outcomes. Furthermore, this serves as an early guide for the prediction of clinical course and deciding management options in a resource‐limited country like Nepal. Further multicenter studies correlating PCT trends with microbiological and clinical outcomes are required to validate the role of PCT levels in postoperative care.

## Author Contributions

**Prati Badan Dangol:** conceptualization, investigation, writing – original draft, methodology, validation, writing – review and editing, visualization, data curation, supervision, project administration. **Battu Kumar Shrestha:** conceptualization, investigation, writing – original draft, methodology, validation, visualization, writing – review and editing, project administration, data curation, supervision. **Suraksha Dhungana:** conceptualization, investigation, writing – original draft, methodology, validation, visualization, writing – review and editing, project administration, data curation, supervision. **Gaurab Mainali:** writing – original draft, methodology, validation, writing – review and editing, software, formal analysis, project administration, data curation, supervision. **Madanika Ghimire:** writing – original draft, methodology, validation, writing – review and editing, data curation, project administration, supervision. **Amrita Pandey:** methodology, validation, writing – review and editing, project administration, data curation, supervision, writing – original draft.

## Funding

The authors have nothing to report.

## AI Disclosure

ChatGPT (OpenAI) was used for grammatical correction and language consistency in the preparation of this manuscript. All scientific content, interpretation, and conclusions were reviewed and verified by the authors.

## Ethics Statement

Ethical clearance was obtained from the Institutional Review Committee (IRC), Shahid Gangalal National Heart Centre (SGNHC), with IRC number: 3‐2023 for the study.

All authors have read and approved the final version of the manuscript. Prati Badan Dangol had full access to all of the data in this study and takes complete responsibility for the integrity of the data and the accuracy of the data analysis.

## Conflicts of Interest

The authors declare no conflicts of interest.

## Transparency Statement

The lead author, Prati Badan Dangol, affirms that this manuscript is an honest, accurate, and transparent account of the study being reported; that no important aspects of the study have been omitted; and that any discrepancies from the study as planned (and, if relevant, registered) have been explained.

## Data Availability

The data that support the findings of this study are available from the corresponding author upon reasonable request.
